# The Quality of Data on Participation in Adult Education and Training. An Analysis of Varying Participation Rates and Patterns Under Consideration of Survey Design and Measurement Effects

**DOI:** 10.3389/fsoc.2019.00071

**Published:** 2019-11-14

**Authors:** Sarah Widany, Johannes Christ, Britta Gauly, Natascha Massing, Madlain Hoffmann

**Affiliations:** ^1^Department System and Politics, German Institute for Adult Education - Leibniz Centre for Lifelong Learning, Bonn, Germany; ^2^Department of Survey Design and Methodology, GESIS – Leibniz Institute for the Social Sciences, Mannheim, Germany; ^3^Division of Further Education and Educational Management, Department of Education and Psychology, Freie Universität Berlin, Berlin, Germany

**Keywords:** adult education and training, adult education and training statistics, survey research, educational monitoring, total survey error, adult learning

## Abstract

Statistics on adult education and training (AET) are often considered as insufficient because they fail to deliver a comprehensive and consistent picture of this field of education. This study addresses a specific problem in AET statistics that is varying participation rates of adults in AET depending on underlying data sources. We elaborate potential causes for deviations in survey design and the measurement of participation in sample based AET statistics with reference to the Total Survey Error (TSE) approach. Our analysis compares AET participation rates and patterns from four representative German surveys and reveals substantial differences in participation rates and mixed results for patterns of participation in AET. We find similar relationships for the influence of employment and educational attainment. The relationship with region, gender, and age shows to some extent deviations that conclude in contradictory statements on probabilities of participation. The discussion addresses consequences for the interpretation of survey results on AET participations and draws conclusions for the further development of AET statistics.

## Introduction

Evidence-based education policy making, nationally and internationally, heavily depends on statistics. Although the databases' quality are crucial, those for lifelong learning are lacking. The concept of lifelong learning expands the traditional focus on learning in the system of initial education to educational activities of adults within their employment, occupational career, and leisure time. Unlike the educational fields of schooling, vocational, and higher education, these lifelong learning activities take place in a plural and heterogeneous training sector for which official statistics are scarce. Instead, representative surveys provide research and monitoring of adult education and training (AET) with data on the context of adults organized learning (e.g., within employment, employers and job characteristics or provider of AET), access and opportunity structures (e.g., according to qualification level, age, regional factors), characteristics of the learning activity (e.g., duration, funding, certification) and individual and wider benefits of AET participation (e.g., income, job security, job, life satisfaction). Unequally distributed AET participation opportunities most notably in favor of those with high skills and favorable employment conditions are a dominant focus in participation research and education policy. AET participation levels is a key indicator and benchmark in educational monitoring (e.g., OECD, 2018, p. 134–146; EU, 2009, p. 119/7; BMBF, 2013) and questions on individuals' AET participation are usually asked as a standard as far as surveys are AET related. Interestingly, the results on AET participation differ considerably. The representative participation rate in AET varies to an extent of 30% points across surveys (Kuper et al., 2016, p. 10). This raises concerns about the quality of AET statistics and consequently where “true” AET participation values lie.

Because little is known about the causes for and consequences of varying AET participation rates across surveys we intend to narrow those gaps with this study and thus contribute to providing more reliable data on AET. We use the Total Survey Error approach (TSE) as a conceptual framework to systematize potential factors for deviations, related to either AET representation or measurement and work with four different representative surveys for Germany that include questions on AET participation. After harmonizing samples and variables, we apply descriptive statistics and multivariate models to illustrate residual variations between the four surveys in relation to AET participation rates and structures. The discussion considers consequences for educational research and monitoring and points out further needs for research as well as possible advancements in AET statistics.

## Causes for Varying Adult Education and Training Statistics—Conceptual Background and Empirical Findings

This chapter introduces the TSE approach in order to explore and systematize potential causes for varying AET participation rates between surveys as well as related empirical findings. It concludes with the elaboration of the research question.

### The Total Survey Error

The TSE Approach provides a paradigm for the quality assessment of surveys in general (Groves and Lyberg, 2010, p. 849). Within a “nested taxonomy” (Groves and Lyberg, 2010, p. 857) a variety of concepts introduce a range of content or process related errors in surveys, which may result in a systematic deviation of a measured value from a true population value, emphasizing different error sources and offering different typologies (Groves and Lyberg, 2010, p. 849). Errors in the TSE paradigm must not be interpreted as mistakes in the colloquial sense, rather they are inevitable or even inherent to the survey process. They hint to central factors that can help to assess and improve survey design (Groves et al., 2009, p. 40f).

**Figure 1:**
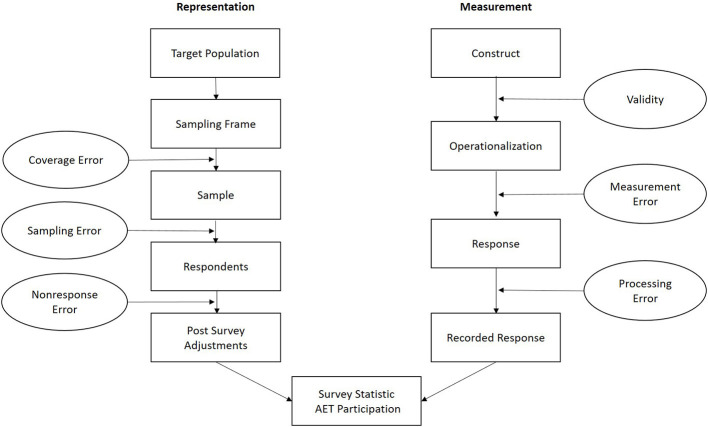
AET surveys from a quality perspective, following Groves et al. (2009) (Figure 2.2, p. 42 and Figure 2.5, p. 48).

For the purpose of this paper, we refer to the established concept of Groves et al. (2009, p. 41–60) and Groves and Lyberg (2010, p. 856f.), that systematizes errors of representation and errors of measurement along the inference process in sample based survey statistics (see Figure 1). Errors of representation include coverage error, sampling error, and non-response error, and emphasize the quality of sampling as a source for varying AET statistics. Errors of measurement, in line with psychometric measurement theories, cover errors related to the (construct) validity of measurement, including errors in data processing and highlight how the quality of measurement might explain varying AET statistics.

A number of error sources align along these two strands. In this chapter, we take a closer look at errors that have been discussed as potential error sources in previous research on AET statistics (see section The Quality of Information From Adult Education and Training Surveys—Current State of Research and Elaboration of the Research Question). In addition to these error sources, we select error sources on which we have information either through the data source itself or via quality reports in the documentation. Further, we focus on bias-related errors, rather than variance-related errors. While the latter are a natural constituent in sample based estimations of population parameters, bias-related errors are not at random and systematically affect sampling or measurement (Groves et al., 2009, p. 56), e.g., excluding respondents with specific characteristics or provoking specific response behavior.

#### Errors of Representation for Varying Adult Education and Training Statistics

Errors of representation result in the misrepresentation of the *target population* in the realized survey sample (Groves et al., 2009, p. 44). Of course, with regard to varying participation rates, differences in the definition of the target population are an obvious explanation. Representative surveys on AET typically target the national household population within a variable age range. Further discrepancies may relate to the inclusion of respondents living in institutional setting (e.g., military, prisons). Moreover, literacy in the survey language (or languages if translations are used) is another characteristic of the target population. Ultimately, the population is always defined by the time of conduction of the survey.

The *sampling frame* is a list from which a random sample of respondents from the target population can be drawn (Groves et al., 2009, p. 54). High quality population registers are considered best practice in survey research. If their quality is not sufficient or if they are not available, geographical listings and random route procedures are applied alternatively (Häder, 2016, p. 8–10; Scherpenzeel et al., 2017). Random route procedures, however, can deliver biased estimations in variables related to the location of the household (Bauer, 2016) as they can systematically favor starting points and routes. Educational opportunities and attainment show significant regional and even local disparities (Ballas et al., 2012; Martin and Schrader, 2016). Additionally, the distance to institutions of AET is related to socio-spatial patterns of participation in AET (Schemmann and Feldmann, 2008). Thus, a biased random route sample might lead to biased estimates on participation in AET. Altogether, the probability of selection for units within the sampling frame can vary according to different modes of sampling. As surveys rarely apply a simple random probability sampling, elements of stratification or clustering are common. Ideally, the non-zero chances of selection into the sample are known for each respondent in the sampling frame (Groves et al., 2009, p. 57ff.) and over- or underrepresentation in the sample can be adjusted in post-survey procedures (see below).

*Non-response* is not easily predictable and can be another source for misrepresentation of units from the target frame. Non-response is a common and increasing phenomenon in survey practice (Groves, 2006). Non-response rates show the percentage of units from the target frame not contacted successfully or which refused to participate in the survey. If non-respondents and respondents differ systematically on the measures of variables essential to the survey (e.g., participation in AET and related variables), survey results are biased by non-response and generalizability is restricted. Low non-response resp. high response rates are therefore often perceived as a quality criterion. However, the degree of non-response does not necessarily say something about the risk of non-response bias (Groves, 2006; Davern, 2013). Low response rates *per se* are not problematic if the refusal does not relate to the survey characteristics. Further, to some extent non-response can be adjusted in postsurvey procedures with information from auxiliary variables (see below; Stoop, 2005, p. 32–36). Furthermore, due to a high variability in the definition and calculation of non-response, (non)response rates are a non-reliable indicator for the comparable assessment of surveys (Davern, 2013, p. 906). Rather than fixation on non-response rates, survey research calls for substantial analyses of non-response bias (Groves, 2006; Groves and Peytcheva, 2008; Davern, 2013), guided by the key question of potential causes for the correlation of survey variables and the likelihood to respond (Groves and Peytcheva, 2008, p. 169). Influencing factors in this relationship are related to characteristics of the survey design (e.g., incentives, sponsorship, mode of data collection, panel attrition, topic), specific survey measurement (e.g., objective vs. subjective measurement, topic interest) and the target population. Most models assume interdependent relationships between these factors (Groves and Peytcheva, 2008, p. 168–170). Altogether, the phenomenon of non-response bias is complex and specific to characteristics of the respective survey and its context, with limited options to draw general conclusions from non-response bias analysis.

Nevertheless, non-response bias related errors point to a range of characteristics as a possible source for varying AET participation rates. Failed representation of the target population in characteristics related to AET participation is a first clue. AET participation shows a pattern in favor of the highly skilled and employed population, amongst others (Desjardins, 2011, p. 209; Gauly and Lechner, 2019). If respondents and non-respondents differ in these characteristics, the estimate of AET participation can be biased. An underrepresentation of low educated and an overrepresentation of higher educated respondents, for example, could entail an overestimation of participation in AET. A comparison of the sample and the population using an external source of population data can address non-response bias at least partially, especially if the comparison variables are central predictors in the model of analysis (Halbesleben and Whitman, 2013, p. 924–925). Interest in the survey topic could be another factor that influences the decision to cooperate in a survey, although literature so far provides mixed evidence (Groves et al., 2004, 2006; Groves and Peytcheva, 2008, p. 184). For AET surveys, this raises the question, if the topic adult education and training is prevalent in the decision process and if this knowledge influences respondents' cooperation. If this is the case, topic interest in AET might suggest some kind of engagement and therefore a higher likelihood of participation in AET, whereas non-interest suggests no engagement and therefore a lower likelihood of participation in AET.

Surveys regularly apply *postsurvey procedures* in order to minimize coverage, sampling, and non-response errors (Groves et al., 2009, p. 59–60, 347–354). Weighting factors calculate factors for every respondent based on their probability of being in the sample. They can account for selection probabilities with regard to sample design (transformation from household to individual sample, stratification) and can redress the sample to known parameters of the target population, e.g., regional and socio-demographic factors, which are known from an external source, such as census data. Surveys commonly use this population-based adjustment, even though the relationship of adjustment variables, response propensity and the measure of interest is not clear and might not necessarily result in a reduction of non-response bias (Peytcheva and Groves, 2009).

#### Errors of Measurement for Varying Adult Education and Training Statistics

Measurement errors bias the true values in answers from survey respondents. A construct of interest, in our case participation in adult education, is the starting point for the development of specific questions that gather information on the construct (Groves et al., 2009, p. 41–43). In contrast to behavioral or attitudinal concepts, participation in AET is not an abstract object of measurement. However, some features can challenge the *operationalization* and *validity* of measurement. There is no established definition to guide the specifications for measurement; the field of AET is quite heterogeneous with regard to participants, providers and formats of learning. It comes with a broad range of concepts and terms (Tight, 2002). Quite obviously, if the underlying concepts of AET and their corresponding question designs vary, we would expect the corresponding participation rates to vary as well. But, even if surveys refer to the same definition of AET, there are no standardized questions to measure them.

Thus, when comparing AET surveys, the exact wording of the question is important. This is where a significant measurement error comes into play, namely the response behavior of respondents (Groves et al., 2009, p. 217–254). In order to comprehend response behavior, survey questions can be examined to determine which cognitive understanding, retrieval, evaluation, and reporting processes they trigger or do not trigger in respondents. From the extensive literature on these complex processes, and their significance for measurement errors (Alwin, 2014), we pick out some aspects that are particularly central to errors in measuring participation in AET.

The *interpretation* of survey questions (Groves et al., 2009, p. 226–229) is central because—as mentioned above—with regard to AET it cannot be assumed that there is a uniform use of language and a uniform universe of terms. If there is no common understanding of AET in the scientific community, a common understanding in the general population is highly unlikely (Widany, 2009, p. 103). Even supposed everyday terms, such as seminar or workshop, which are typically used in questions on AET participation, can be interpreted very differently at the respondent level (see below). Furthermore, questions about AET participation relate to the past and respondents must *recall* the events (Groves et al., 2009, p. 229–234). Whether this recall is successful depends on several factors (Dex, 1995), among others, on whether the terms used in the survey match those of the respondents. Individuals may have taken part in a weekend seminar, but do not associate this with the terms “participation in training” used in the questionnaire. Further aspects that can influence the quality of recall are the proximity to significant temporal boundaries, such as biographical life events, the emotional significance of events and their temporal proximity or distance to the interview. The *questionnaire design* can account for some of these factors, e.g., referring to a reasonable period for recall or individual significant time periods. Examples of AET activities and a broad use of related terms (supported by visual aids such as listings on show cards) is likely to trigger memories on AET events and improve recall. However, this increases the complexity of the question and thereby the cognitive efforts to evaluate and report to it. If respondents report on AET participation by third parties in proxy-reports, e.g., for household members, this recall acting on behalf of another person may be less accurate than if this person had been interviewed him- or herself (Groves et al., 2009, p. 246).

Survey research in panel studies further gives some hints, that panel participation influences respondents' response behavior by *panel conditioning* in two ways. As they grow familiar with the questionnaires structure and interview situation, they can report more accurately or they learned where to report false answers in order to skip questions and reduce the length of the interview (Warren and Halpern-Manners, 2012). Apart from response behavior and associated measurement errors, some studies show that participation in panel surveys can influence the behavior of respondents in the surveyed topics, as the survey raises the awareness and motivation to become more active in a particular topic. For example, in a panel survey on labor market issues, repeated questions on participation in labor market programs result in respondents being more likely to participate in these programs than non-surveyed control groups (Bach and Eckman, 2019).

Furthermore, in surveys with *face to face or telephone interviews*, the behavior of interviewers affects survey errors in two ways. First with regard to representation, their first contact with potential respondents can influence the respondents' decision to cooperate in a survey or not (non-response, see above). Second with regard to measurement, they can influence respondents' answers in the course of the interview directly through the administration of the questionnaire or indirectly through their characteristics and behavior in the social situation of an interview (Groves et al., 2009, p. 291–324; Brunton-Smith et al., 2017). As a result, survey estimates can be dependent on who conducted the interview. To reduce interviewer effects, surveys can reduce the number of interviews per interviewer in order to minimize the impact of the individual interviewer on the total sample. Furthermore, sound interviewer training and interviewer experience warrants high standards in the administration of the questionnaire and supports good answers from respondents.

### The Quality of Information From Adult Education and Training Surveys—Current State of Research and Elaboration of the Research Question

Overall, the state of research on the quality of information on AET participation is manageable and concentrated in countries that implemented AET strategies and monitor them with available AET statistics. In general, there is a perception that the existing data base on AET is insufficient and “poor training statistics” (Felstead et al., 1997, p. 12; Kuper et al., 2016, p. 10–12) need to be improved, with regard to both information and data quality. Analysis either have a primary focus on one or several AET statistics and assess their quality or take up the topic in connection with the suitability of secondary data for analysis projects. If possible, comparative studies usually harmonize sample characteristics (e.g., age span, employment status) and measurement (e.g., exclusion of specific AET activities) and thereby exclude these features as explanatory factors for differences in AET participation. Usually, the quality of surveys is assessed in terms of both representation and measurement errors. Findings from cognitive interviews or language use studies provide further contributions with regard to the measurement of AET.

The Adult Education Survey (AES) and the Labour Force Survey (LFS) are typically addressed as Europe-wide surveys as well as nationally specific surveys. The comparison of sample characteristics from voluntary surveys, e.g., the AES, to those of surveys with a huge sampling rate or a compulsory participation (one or the other applies, for example, to the LFS in most European countries), shows deviations in the direction of an overrepresentation of high educational attainment and occupational positions in the voluntary surveys (Lois, 2005; Wohn, 2007; Widany, 2009, p. 141; Badescu et al., 2013). This could be an indication of a systematic non-response. Both, educational attainment and occupational position highly correlate with participation in AET, therefore, participation rates can be overestimated in the voluntary surveys. Authors also discuss a non-response bias that relates to an interest or engagement in the topic of AET (Wohn, 2007; Widany, 2009, p. 143; Eisermann et al., 2014). However, available data does not facilitate further analysis of non-response. Interestingly, none of the available studies includes further quality criteria that can influence the representativeness of the results, e.g., sampling design, response rates, or weighting procedures, in order to discuss deviations in AET participation rates.

Varying measurement of AET is often considered the most important cause for varying AET participation rates. A study on language use demonstrates that overall, respondents understanding of AET is rather narrow and the interpretation of the meaning varies with educational background and other characteristics (Campanelli and Channell, 1994). Comparative studies find that higher participation rates in surveys come hand in hand with a more exhaustive measurement of AET participation, including different training activities and examples, supported by listings and show cards (Wohn, 2007; Eisermann et al., 2014). Indeed, presenting a listing of a range of training activities to respondents who reported no participation in AET in the first place helped respondents to recall activities (Felstead et al., 1997, p. 32). Yet, the comparison of surveys with relatively similar measurement of AET still reveals substantial variations in participation rates (Lois, 2005; Widany, 2009, p. 142–143). Similar to the assumptions about a systematic non-response (see above), it is discussed in this context whether the thematic range of the survey is related to respondents propensity to report AET participation, with one-topic surveys on AET attracting more AET active respondents than multi-topic surveys with a potentially more diverse sample (Wohn, 2007; Widany, 2009; Eisermann et al., 2014). The length of the reference period for which AET activities are surveyed influences reports on AET participation in two ways. First, as AET is usually not an everyday activity, longer periods of reference increase the likelihood of AET participation. An extension from a period from 4 to 13 weeks can significantly boost participation rates (Felstead et al., 1997, p. 31–32). Differences in length of reference period are regarded as the main factor for disparities in the level of AET participation between AES (12 months) and LFS (4 weeks) (Badescu et al., 2013). Second, an extended reference period increases recall problems. A cognitive pretest showed that a context based question design (e.g., participation in AET in the context of a specific employment), supports respondents' accurate recall within the last 5 years (Dürnberger et al., 2010). Leveling reference periods and aggregated participation rates does slightly adjust participation levels between surveys (Widany, 2009, p. 124; Eisermann et al., 2014). The use of proxy interviews seems to undercover participation in AET as self-reports show higher levels of AET participation than proxy-reports. On the one hand, proxied respondents show characteristics that are related to higher likelihood of AET participation (young and male). On the other hand, proxy reports on AET activities of a third party are likely to be less accurate (Felstead et al., 1997; Lois, 2005; Wohn, 2007). A multivariate analysis controlling for several determinants of AET shows a negative relationship between participation in AET and proxy interviews (Blundell et al., 1996).

In brief summary, the sparse literature primarily discusses differences in the measurement of AET as a cause for varying participation rates, emphasizing the level of support for interpretation and recall of the questionnaire design, the reference period and the undercoverage by proxy interviews. With regard to representation, the bias of samples from voluntary one-topic surveys toward characteristics that increase the likelihood of AET participation is discussed. However, the reference to specific survey features is not always systematic and hardly does justice to a comprehensive perspective. Findings are usually based on descriptive comparisons and do not permit statements of causal relationship.

Participation in AET is an important global indicator and descriptive statistics give us valuable information on participation (for different groups) by means or averages. In addition to the extent of participation, other qualitative aspects are of interest. For example, the question of equal opportunities for participation in AET requires an analytical approach that sheds light on the associations of participation with different attributes. Generally, with regard to survey errors, the analytical use is prone to the same errors as is the descriptive use, but might affect analytical results differently. If, for example, AET participation is underreported in all qualification groups the descriptive results are not accurate. Nevertheless, the association between qualification and participation in AET is not affected, as far as underreporting is consistent over all qualification groups. However, if AET participation is underreported by specific qualification groups only, the association is biased (Groves et al., 2009, p. 61–62). The few findings from the literature rather indicate that not only distribution parameters, but also statistical relationships vary between the surveys. Analyses using the same models over different survey statistics show, that a sample bias toward higher educated and occupational superior older age groups can mitigate an otherwise strong negative effect of age on AET participation (Lois, 2005). Apart from the strength of relationships, findings eventually point to deviations concerning the direction of the relationship, e.g., a positive relationship with unemployment in one and a negative or not significant relationship in the other survey statistics (Wohn, 2007; Badescu et al., 2013). However, analyses partly use variables for predicting AET participation for which comparability between surveys is just as problematic as the measurement of AET, and they refer to different survey years. Therefore, it is hard to tell, whether the relationship changed over the course of time, or is biased by the incompatible measurement of other determinants. This calls for further research on the quality of analytical findings on AET participation patterns based on different AET surveys.

We ask from a TSE perspective about distinct features in survey design and measurement in population based AET surveys as a possible source for varying participation rates. Upon this, we extend the comparison of participation rates to a multivariate analysis of AET participation patterns in order to evaluate the descriptive and analytical use of data from different AET surveys. Since research and reporting often focus on vocational AET, we consider overall participation and participation in vocational AET separately.

## Comparative Analysis of Adult Education and Training Participation and Participation Patterns in Four Population-Based Surveys

In general, all surveys that cover participation in AET could be considered as eligible for our analysis. However, drawing conclusions about the impact of distinctive survey features on AET participation rates and patterns in a comparative analysis requires a minimum of a comparable basis. The selection process resulted in four surveys, which are all based on representative samples of the German population from an overlapping reference period (roughly year 2012) and serve as sources for educational monitoring as well as scientific research. The Adult Education Survey (AES) (BMBF, 2014) and the Programme for the International Assessment of Adult Competencies (PIAAC) (Rammstedt et al., 2016) are both international comparative surveys within the EU, respectively OECD-countries. In comparison, the AES has the most comprehensive AET-related information and provides detailed information on informal, non-formal and formal adult learning, as well as attitudes and barriers to learning. PIAAC also covers educational activities, but focuses on adults' skill measurement in literacy, numeracy and problem-solving in technology-rich environments. The Microcensus (MC) (RDC, 2012) is the official statistic of the German population and its economic activity and integrates the European Labour Force Survey (LFS). The MC aims at describing the population as broadly as possible on a yearly basis and AET is one of many areas of characteristics surveyed. The National Educational Panel Study (NEPS) (Blossfeld et al., 2011) provides longitudinal data on educational trajectories and processes for different birth cohorts. The NEPS measures participation in AET embedded in (employment) biographies of adults. All four surveys have in common that they continuously collect data on AET and cover a similar time period. They are considered the most relevant data sources for AET in Germany. They have a similar target population and a similar definition of AET. As a panel study, NEPS' primary purpose is not the provision of cross-sectional estimates and therefore not an ideal comparative subject. Nevertheless, we included it in the comparison, as it is a relevant data source in German AET research and the measurement of AET participation in particular is an interesting addition to the approach of the other surveys. The following subsections further elaborate common and distinct features. For systematization, we again use the distinction between characteristics related to representation and measurement errors.

### Comparison From the Perspective of Representation Errors

Table 1 gives an overview on the specifics of the surveys^1^. The detailed comparison shows that, in addition to basic similarities, there are variations in numerous characteristics that can in principle be the cause of deviations in the AET estimates. What they have in common is government sponsorship and long-term operation by established survey institutes or official statistical offices. The fieldwork of all surveys is in 2012, and partly extends into the previous or the following year. The data collection mode is mostly the Computer Assisted Personal Interview (CAPI), also within the mixed-mode surveys, which also apply Computer Assisted Telephone Interview (CATI; in NEPS) and additionally self-administered paper questionnaires (in MC). The age range of the target population varies over the surveys. With the exception of the MC, all surveys exclude persons in shared accommodation from the definition of the target population. For sampling, all surveys apply some variation of (multilevel) stratification and cluster procedure; all except AES select the target persons from population registries. AES is the only survey with a random route procedure. The mandatory participation in the MC results in an extremely high response rate, which is why unit non-response errors should not be a factor. The NEPS also stands out with a relatively high response rate. As this wave only includes panelists and no first respondents it rather indicates panel attrition of respondents from previous waves. This panel attrition is likely to be subject to a different mechanism compared to the non-response in the voluntary surveys, which in the case of AES and PIAAC is around 50% each. The number of successful interviews (sample size) ranges from ~5.500 (PIAAC) to 698.000 cases (MC). From the voluntary surveys, NEPS and PIAAC used recruitment strategies to motivate respondents to participate in the survey. The AES contacts respondents without prior notice at their residence. Interestingly, information on the topic is only presented after the respondent asks what the survey is about. How often respondents inquire about this and whether this relates to non-response behavior could shed some light on the relationship between non-response and topic interest. However, there is no data available. It is likely that the surveys differ in the way interviewers were engaged, as interviewer training is only reported for NEPS and PIAAC. Both carry out elaborate competence measurements, which certainly place special demands on the administration of the interviews. The weighting procedures for all surveys apply design and redressment adjustment. There is a core of similar redressment parameters and data sources and some survey specific ones [e.g., country of birth (NEPS), citizenship (AES and MC)]. All but the AES indicate that their weighting takes into account results from non-response analyses.

**Table 1:** Overview of survey design of AES, PIAAC, NEPS, and MC.

	**AES**	**PIAAC**	**NEPS (SC 6, wave 5)**	**MC**
Sponsorship	Government	Government	Government	Government
Survey Institute	Infratest (since 2016 known as Kantar Public)	Infratest (since 2016 known as Kantar Public)	Infas	Statistical offices of the States (Länder)
Study design	Cross-sectional study	Cross-sectional study	Yearly panel-study	Cross-sectional study, rotating panel
Data collection method	CAPI	CAPI	CAPI (87.5%), CATI (12.5%)	CAPI (76.8%)/self-administered paper questionnaire (20.8%)/CATI (2.4%)
Fieldwork	03/2012–06/2012	08/2011–03/2012	10/2012–05/2013	All through the year 2012
Target population	Residential population, living in private households in Germany, aged 18–64	Residential population, living in private households in Germany, aged 16–65	Residential population, living in private households in Germany, birth cohorts 1944–1986	Residential population, living in private households, and shared accommodations in Germany aged 0 and over (AET: aged 15 and over)
Language	German	German	German, Russian, Turkish	German
Sampling frame and design	Stratified multilevel random sample, random route procedure, selection key for respondent in household	Population registry-based, two-stage stratified and clustered random sample	Population registry-based, stratified multilevel random sample	Population registry-based single-stage stratified cluster sample; yearly replacement of one quarter of the sampling units
Sample size	7,099 respondents, 16,322 target persons (gross sample size)	5,465 respondents, 10,240 target persons (gross sample size)	11,696 respondents, 15,249 target population	688,900 respondents; scientific use file covers 70% of the sample (511,946 including 476,342 principally residents)
Obligation	Voluntary	Voluntary	Voluntary	Mandatory
Response rate	49.7%	55% (incl. design weights)	76.7%	97.9 % of households
Recruitment /contact	Doorstep	Advanced letter, monetary incentive	Advanced letter, monetary incentive	Official notice in advance
Survey is introduced as	…a scientific study; topic “adult learning” is introduced upon respondents' request	…a study on adults skills	…a scientific study on adults education and lifelong learning	…survey on the population and the labor market
Interviewer	No information	5-day-training,	1–5-day-training	No information
Weighting procedure	Design adjustment and redressment to parameters of official statistics	Design, non-response adjustment and redressment to parameters of official statistics	Design adjustment and redressment to parameters of official statistics	Non-response adjustment and redressment to parameters of official statistics
Sources; Quality reports	Bilger et al., 2013; Infratest, 2013	Rammstedt, 2013; Zabal et al., 2014	Blossfeld et al., 2011, 2016; Bech et al., 2013; Hammon et al., 2016; FDZ-LIfBi, 2019	Destatis, 2013

*Abbreviated version. Table S1 provides more details*.

### Comparison From the Perspective of Measurement

We draw the information on measurement of AET directly from the questionnaires of the surveys and consider the items as the empirical implementation of an underlying construct of AET. Conceptually, the definition of adult education and training in the four surveys follows the internationally established Classification of Learning Activities (CLA) by Eurostat (2016). The CLA distinguishes formal education, non-formal learning and informal learning. Non-formal education is defined as “education that is institutionalized, intentional and planned by an education provider. The defining characteristic of non-formal education is that it is an addition, alternative and/or complement to formal education [*that leads to a recognized qualification in the national education system, often initial education, S.W*.] within the process of lifelong learning of individuals […]. It caters to people of all ages but does not necessarily apply a continuous pathway structure; it may be short in duration and/or low-intensity; and it is typically provided in the form of short courses, workshops or seminars […]” (Eurostat, 2016, p. 15). Overall, all four studies follow this definition of AET. In particular, they all point out that AET is a form of learning that takes place in addition or as a complement to formal education and is distinct from informal learning, that is intentional but less structured.

Table 2 gives more details on the measurement of AET in the surveys. All surveys provide a variety of terms and occasions to guide respondents' interpretation of AET and support the recall of AET events, both in the introductory question as well as with help of show cards and examples. Judging by the number of terms and examples used, interpretation and recall of AET is likely to be more stimulated in AES and PIAAC than in NEPS and MC. Some terms and related activities (e.g., courses, training, seminars, private lessons) are cited in all surveys, others only appear in individual surveys [e.g., lectures (AES), distance learning (PIAAC), retraining (MC)]. Only AES and PIAAC mention work-related training/courses by colleagues or supervisors explicitly. Only MC includes conferences in its list of AET formats, which is usually, and according to CLA, classified as informal learning. For recall, AES, MC, and PIAAC refer to an identical period of 12 months preceding the interview. NEPS supports respondents' memory as they ask for AET participation within specific contexts, such as recent or current employment, parental leave, or retirement episodes. If the number of reported activities exceeds a specific number, all surveys but MC apply a selection process in order to ask detailed questions on the selected AET activities. These details also include the information used to classify the activities as vocational or non-vocational, respectively attending a course for professional or private reasons (see Table 3 and section Data Preparation—Enhance Comparability in Key Variables and Samples).

**Table 2:** Overview of measurement of AET in the AES, PIAAC, NEPS, and MC.

	**AES**	**PIAAC**	**NEPS (SC 6, wave 5)**	**MC**
Introduction to AET section in the interview addresses…	Wide range of AET opportunities Classification of AET activities	No specification: other courses and training (previous section is about formal education)Classification of AET activities	Courses and seminars attended in specific episodes and outside episodes	Straightforward: Have you participated in general or vocational training in the last 12 months?
Reference period AET participation	12 months previous to interview	12 months previous to interview	Time span since the last interview	12 months previous to interview
Support of recall	Show card with a list of four kind of activities [each with specific vocational or non-vocational examples] Courses and training courses in work or leisure Short-term educational events: Lectures, training courses, seminars, workshops Training/on-the-job training by supervisors, colleagues, trainers, teletutors Private lessons in leisure time	Show card with a list of four kind of activities Courses for professional or non-professional reasons Course conducted through open or distance education Organized session for on-the-job training or training by supervisors or co-workers Seminar or workshop Other kind of course or private lesson	Recall within specific contexts Now I have a few questions about the courses and seminars you have attended during <for example civilian service, this activity, parental leave, ...> last year Reference to courses “made for yourself,” e.g., by attending a cooking, language or a trainer course	Further explanation: for example courses, seminars, training courses, conferences, private lessons, study circles Definition vocational AET: retraining, courses for professional advancement, new professional tasks, further education Definition non-vocational AET: courses for private purposes, acquire, or expand one's own skills and knowledge
Number of activities and selection process for inquiry on further details	12 AET activities, further loops for up to 4 and up to 2 (randomly selected) activities	1 AET activity, most recent	2 (randomly selected) AET activities	Global questions for all resp. Last activity reported
Panel conditioning	First time respondents	First time respondents	Panel respondents	First time (25%) and panel respondents (75%)
Placement in questionnaire (roughly)	In the middle of the interview	At the beginning of the interview	Mainly within reported episodes, varies over respondents	Toward the end of the interview
Proxy interviews	No	No	No	26%
Original questionnaire (German version)	Infratest, 2013	Gesis, n.d.	LIfBi, 2016	Statistische Ämter des Bundes und der Länder, 2012

*The questions are translated using the online machine learning translation service DeepL (https://www.deepl.com/translator [translation on the dates July 18th to 19th]) to ensure a certain degree of standardization and objectivity; this table shows keywords and phrases. We provide an extended version in Table S2*.

**Table 3:** Operationalization of vocational AET in the AES, PIAAC, NEPS, and MC.

	**AES**	**PIAAC**	**NEPS (SC 6, wave 5)**	**MC**
Information to identify vocational AET from up to	12 AET activities	1 AET activity, most recent	2 (randomly selected) AET activities	Global questions for all activities reported
Measurement of vocational AET	Mainly for professional reasons or more for private reasons?	Mainly for professional reasons?	For professional or private reasons?	Purpose of your training?
Values^a^	**1** **=** **professional**; 2 = private	0 = no; **1** **=** **yes**	**1** **=** **for professional reasons**; 2 = for private interest; **3** **=** **both**^b^	**1** **=** **professional**; 2 = private; **3** **=** **both professional and private**
Activities automatically assigned to vocational AET	Training/on-the-job training by supervisors, colleagues, trainers, teletutors	Organized session for on-the-job training or training by supervisors or co-workers		

a *Bold values are included in the definition of vocational AET*.

b *According to the manual, “both” is not read out as an answer category and is rather an option for the interviewer if the respondent answers correspondingly*.

*The questions are translated using the online machine learning translation service DeepL to ensure a certain degree of standardization and objectivity; this table shows heavily shortened excerpts. An extended version is provided in Table S3*.

Apart from the questions, the surveys show distinct features that can affect the quality of measurement of AET: First, the panel design of NEPS and MC might facilitate a familiarity with the questionnaire that either promotes answers that are more accurate or it might provoke item non-response, in order to shorten interview duration by skipping questions. Second, compliance with the survey and cognitive ability to recall can also depend on the duration of the interview and the placement of the questions on AET within the questionnaire. Quality of recall and willingness to report correctly can decrease with the progress of the interview. A third feature that probably affects the assessment of AET are proxy interviews, which are only present in the MC (~26% of the interviews). Here, respondents also answer questions for other household members, which probably affects reported participation rates.

### Empirical Comparison of Adult Education and Training Participation and Patterns

Previous chapters described a range of characteristics of the surveys as potential error sources and causes for varying participation rates in AET from the perspective of the TSE paradigm. Unfortunately, we cannot disentangle the causes and effects in that respect with the available data. It is however possible to give a more comprehensive picture about diverging results on AET participation. Based on harmonized samples, we compare sample characteristics and participation rates in a descriptive analysis and use regression models to predict AET participation to compare analytical results of the four surveys.

#### Data Preparation—Enhance Comparability in Key Variables and Samples

The data was prepared according to the highest common denominators. All surveys approximately refer to the reference period of 2011/2012. The period for which AET is reported, is the first and the last individual interview date during field work (see Table 1) minus 12 months. As the MC is conducted throughout the year, the reference period for participation in AET in the MC 2012 is a rolling period over (roughly) 2 years from January 2011 to December 2012. The NEPS covers participation in AET since panel members most recent interview (persons who did not participate in the preliminary wave 4 were excluded). As the intervals between interviews vary between 5 and 19 months (mean: 12.15; median: 12.00), so does the reference period for AET participation. The overall period for which AET is reported in NEPS is 10/2011 to 05/2013. For AES and PIAAC the reference period is the first and last interview during fieldwork (see Table 2) minus 12 months (AES 03/2011–06/2011; PIAAC 08/2010–03/2011).

We analyze overall AET participation and vocational AET participation. Analyses of AET usually focus on vocational AET. These job-related participations account for by far the largest share of reported AET activities. The distinction between job-related and non-job-related participation makes sense, since specific motivations, characteristics and returns to participation are assumed in each case (Desjardins, 2015). Table 3 provides further details on the survey specific operationalization of vocational AET. Overall AET participation includes any reported activity in the last 12 months (except for NEPS, see above). Vocational AET participation includes any AET activity for which respondents reported an attendance for professional reasons in a follow-up question. Between the surveys, the number of activities selected for this follow-up question varies, so do the selection probabilities due to different selection criteria (e.g., most recent activity, random selection, or global question referring to all activities). Activities which are very much embedded in the work context (AES and PIAAC) are automatically classified as vocational. In AES and PIAAC, respondents choose between the two options private vs. professional. The NEPS and the MC offer a third category in which both, professional and private reasons are valid. Our coding assigns activities that respondents report as both vocational and non-vocational (NEPS and MC) to participation in vocational AET, although they could just as well be assigned to non-vocational AET according to the same logic. It is difficult to say whether the measurement of AET after narrowing to vocational AET is more comparable than the measurement of overall AET. In terms of content, the reference may be more uniform across the surveys as it further specifies the AET construct and excludes a number of rather heterogeneous and probably strongly varying non-vocational activities over the surveys. However, the described technical peculiarities of the surveys in turn limit comparability.

We restricted the age range in all studies to 25–64 years. This was necessary as the AES does not sample persons over 64 years of age, PIAAC does not ask respondents who are currently engaged in formal education and younger than 25 years about participation in AET and NEPS does not sample persons younger than 25 years of age. Additionally, MC data was restricted to respondents at their main residence resp. respondents at their secondary residence were excluded in order to avoid duplicated observations.

For the analysis of sample characteristics and participation patterns we selected socio-demographic variables with a high comparability over all surveys. The selection was based on the predictors frequently used in research for the analysis of AET participation (e.g., Desjardins, 2011), but was mainly determined by whether these variables were available in a comparable manner across the surveys. The latter was surprisingly rare. Age is shown in age bands of 10 years, gender measured by male or female, region by residence in East or West Germany. Education is reported in ISCED 1997 in 4 different groups (ISCED 1 and 2; ISCED 3A, 3B, and 4; ISCED 5B; ISCED 5A/6). Employment status distinguishes between employed respondents and others. In AES, PIAAC, and NEPS, the employment status is measured according to the subjective status reported by the respondent. This was assessed with help of a list of different statuses, such as full-time employed, part-time employed, unemployed, retired, student etc. In the MC, the analyzed variable refers to the reported employment status within the last week prior to the interview date. In NEPS, to reproduce the cross-sectional logic of the other surveys, the reported employment status in the month of the interview was constructed as employment status.

Each of the data sets we use has a different weighting structure that aims at obtaining a distribution of socio-demographic variables that is representative of the German population. We applied the study-specific weighting procedure in each of our analyses. Weighting factors are calculated based on the full sample. Nature of the secondary data does not allow manipulating weighting factors. Therefore, objective of sample restriction was to minimize manipulation in the original sample and thereby apply the original weights within their intended scope.

#### Methodical Approach

We apply a three-fold strategy in order to compare the measurement quality of AET across data sets. First, we compare the distribution of important socio-demographic variables between our samples. In a second step, we investigate how the participation rates in the four surveys differ for overall AET and vocational AET.

Beyond the comparison of participation rates, we are interested in how differences in survey design and measurement of AET affect estimated participation probabilities. Thus, in our third step, we model the relationship between AET and a set of socio-demographic characteristics by using weighted logit models^2^:

(1) $$\begin{array}{r} \mathrm{Pr}\left(AET=1|X\right) \end{array}$$

The dependent variable is participation in overall AET, respectively vocational AET (*1* = *participation, 0* = *no participation*). *X* is a vector, which includes the following set of covariates:

Region (*2 categories, reference: West Germany*), sex (*2 categories, reference* = *male*), age (*4 categories, reference* = *25–34*), educational attainment (*4 categories, reference* = *ISCED-97-levels 5A and 6*), and employment status (*2 categories, reference* = *employed*)^3^.

We conduct the regression analyses for each of the four surveys separately and calculate average marginal effects (AMEs) and predictive margins (PM). AMEs give the average probability to participate in AET compared to a reference category. For example, an average marginal effect of 0.05 for the gender coefficient (*1* = *female, 0* = *male*) would indicate, that—everything else equal—women are on average five percentage points more likely to participate in AET than men. PMs are calculated for every value of a variable and give the absolute probability to participate in AET. For example, a PM of 0.2 for men indicates that—conditional on the other control variables in the model—men have a 20 percent probability of participating in AET. The relationship between AMEs and PMs is that AMEs express the difference of the PMs for the different values of a variable. In the example above, the PM for women would then be 0.25.

**Figure 2:**
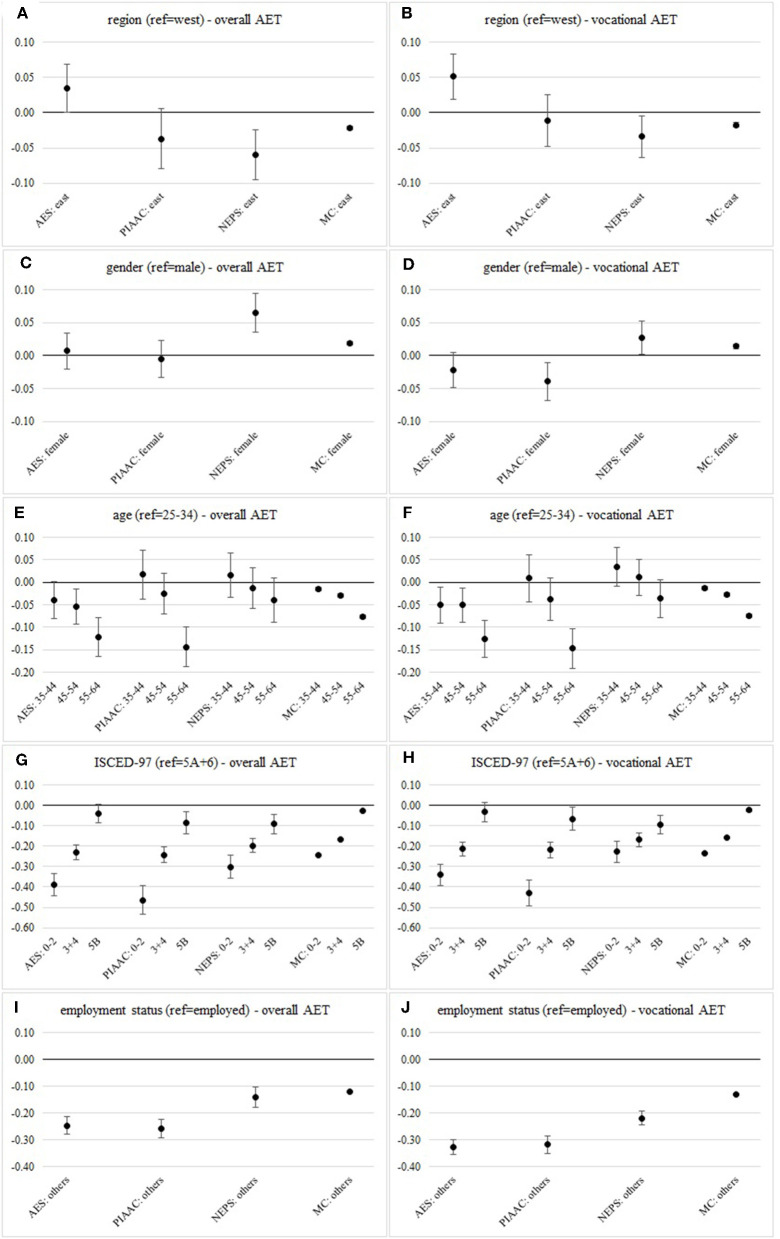
Average marginal effects on the probability to participate in AET in Adult Education Survey (AES), Programme for the International Assessment of Adult Competencies (PIAAC), National Educational Panel Study (NEPS) and Microcensus (MC) of **(A)** region on overall AET, **(B)** region on vocational AET, **(C)** gender on overall AET, **(D)** gender on vocational AET, **(E)** age on overall AET, **(F)** age on vocational AET, **(G)** ISCED-07 on overall AET, **(H)** ISCED-07 on vocational AET, **(I)** employment status on overall AET, and **(J)** employment status on vocational AET. Data sources: AES 2012, PIAAC 2012, NEPS SC 6 wave 5, Microcensus 2012 (weighted, own calculations).

We plot the AMEs for each covariate and each of the studies in Figure 2. In this way, we can directly compare how the differences in the probabilities to participate in AET vary between different socio-demographic groups and surveys. Additionally we provide the calculated PMs in Tables S7, S9 which reflect the differences in the participation rates between the surveys and illustrate the differences in probabilities for the baseline categories.

## Results

Our empirical comparison of the surveys starts with an overview of sample characteristics. Subsequently, we provide details on the participation rates in overall AET and vocational AET and compare the results with and without weighting procedures within the surveys. Finally, the estimated logistic regression models show if and how patterns of participations in overall and vocational AET vary between the surveys.

### Sample Characteristics

Table 4 shows the unweighted and weighted distributions of the socio-demographic variables in our harmonized sample of individuals aged from 25 to 64 years across the four surveys. The comparison provides an idea of how the weighting contributes to sample parameters matching the distributions of the target population and to what extent the representativeness of the sample depends on postsurvey adjustments.

**Table 4:** Sample characteristics of AES, PIAAC, NEPS, and MC.

	**Without weights**				**Weighted**			
	**AES**	**PIAAC**	**NEPS**	**MC**	**AES**	**PIAAC**	**NEPS**	**MC**
Full sample: individuals aged 25 to 64 (N)	6,213	4,350	10,428	258,444	6,213	4,350	10,428	258,444
Region (N)	6,213	4,350	10,415	258,444	6,213	4,350	10,415	258,444
West (%)	77.97	78.71	79.66	79.21	79.57	79.80	78.38	79.77
East (%)	22.03	21.29	20.34	20.79	20.43	20.20	21.62	20.23
Gender (N)	6,213	4,350	10,428	258,444	6,213	4,350	10,428	258,444
Male (%)	47.22	48.69	49.01	49.54	50.46	50.44	51.49	49.97
Female (%)	52.78	51.31	50.99	50.46	49.54	49.56	48.51	50.03
Age (N)	6,213	4,350	10,428	258,444	6,213	4,350	10,428	258,444
25–34 (%)	20.28	22.85	14.59	20.90	21.85	21.44	14.76	21.98
35–44 (%)	24.18	25.84	20.52	23.55	25.86	26.57	23.22	24.61
45–54 (%)	30.66	30.09	37.50	30.64	29.73	29.49	31.83	29.72
55–64 (%)	24.88	21.22	27.39	24.91	22.56	22.50	30.20	23.69
ISCED-97 (N)	6,208	4,268	10,417	257,947	6,208	4,268	10,417	257,947
0–2 (%)	10.53	8.39	5.51	13.27	11.79	9.92	9.27	13.60
3+4 (%)	57.01	53.49	44.77	58.54	57.13	55.54	53.26	58.23
5B (%)	14.06	14.41	20.64	11.35	13.95	13.53	17.81	11.19
5A+6 (%)	18.40	23.71	29.08	16.84	17.13	21.01	19.66	16.98
Employment status (N)	6,212	4,272	10,428	258,444	6,212	4,272	10,428	258,444
Employed (%)	70.41	75.59	84.25	74.36	74.77	74.52	81.95	74.48
Unemployed and others (%)	29.59	24.41	15.75	25.64	25.23	25.48	18.05	25.52
Overall AET (N)	6,213	4,272	10,428	258,156	6,213	4,272	10,428	258,156
Yes (%)	48.19	52.43	40.77	16.71	48.49	50.22	35.32	16.72
No (%)	51.81	47.57	59.23	83.29	51.51	49.78	64.68	83.28
Vocational AET (N)	6,211	4,271	10,062	258,156	6,211	4,271	10,062	258,156
Yes (%)	41.60	45.33	31.27	15.88	42.10	43.52	26.66	15.88
No (%)	58.40	54.67	68.73	84.12	57.90	56.48	73.34	84.12

*AES (2012) and PIAAC (2012); NEPS SC 6, wave 5; Microcensus 2012 (own calculations)*.

Due to the high response rate and large sample size in the MC, there are only minor deviations, most pronounced in the age variable. In comparison, we see slightly more shifts in the AES, PIAAC, and NEPS data. As these three studies use the MC as reference for weighting, we also see that they move toward the distributions of the MC. With respect to educational attainment in AES, PIAAC, and NEPS, the share of higher-qualified respondents (levels 5B, 5A, and 6) decreases toward the share within the MC. This reflects previous findings from the literature that indicate an overrepresentation of high-qualified respondents in voluntary surveys (see section The Quality of Information From Adult Education and Training Surveys—Current State of Research and Elaboration of the Research Question).

Although the sample distributions align, some differences between the surveys remain after the weighting adjustments. Whereas, in AES, PIAAC, and NEPS there are slightly more women than men, the opposite is the case in the MC. There are moderate deviations in the age distributions; the share of the population aged 45–64 years lies in between a range of 52.0 (PIAAC) and 53.4% (MC). Educational attainment varies between all four surveys, which is particularly evident in lower and upper qualification categories. The share of people with low educational attainment (levels 0–2) in AES and PIAAC is still smaller (with difference up to 3.7% points) than in the MC. Simultaneously, in PIAAC the share of academics (levels 5A and 6) is higher in comparison to the MC (4.0% points). The corresponding share in AES is only slightly below MC.

The NEPS data is to some extent an outlier: its distributions differ more strikingly in terms of age (older), education (higher qualified), and employment status (less unemployed). As these differences are probably due to its cohort panel structure and the simulation of a cross-sectional sample for the purpose of our analysis, we do not make any further population-based comparisons with the cross-sectional studies. However, we need to keep in mind that these deviations might affect the aggregated chances of participating in AET (older age negatively, higher qualifications, and employment positively).

### AET Participation Rates

Whereas, the results for socio-demographic characteristics show some shifts in the distributions before and after weighting adjustments, the participation rates (see Table 4) show only minor deviations within AES, PIAAC, and the MC. This applies to both overall and vocational AET. The comparatively strong shifts in NEPS with regard to sample characteristics before and after weighting are continuing in pre and post weighting participation rates; overall and vocational AET decrease by 5.5 and 4.6% points after the adjustments.

In order to meet the representative requirements of the surveys, the following results refer exclusively to the weighted data. We see substantial deviations in the levels of participation rates between the surveys. Between the MC with the lowest and PIAAC with the highest participation rates, there is a gap of 33.5% points in overall AET. In between this range, participation rates in AES (48.2%) are rather close to PIAAC, while the NEPS rate (40.77) lies below PIAAC and AES but still significantly above the MC rate. When focusing on vocational AET activities, the participation rate in the MC decreases by ~5.0%, in the AES by 13.2%, in PIAAC by 13.3%, and in the NEPS by 24.5% of the overall-AET-value^4^. Similar to before, we find the largest gap between the MC rate and the PIAAC rate: 27.6% points. However, for various reasons (see section Data Preparation—Enhance Comparability in Key Variables and Samples) these different decreases in participation rates from overall to vocational AET cannot be interpreted as a direct effect of the operationalization of vocational or non-vocational AET.

After the descriptive comparison of sample characteristics and AET participation rates, we extend the comparison toward participation patterns of AET in order to investigate the analytical use of the survey data.

### Adult Education and Training Participation Patterns

Figure 2 shows the AMEs of our weighted logistic regression models of participation in overall (left side) and vocational AET (right side) on the set of socio-demographic characteristics (also see Tables S6, S8 and in addition Tables S7, S9 for PMs).

Whereas, the results of PIAAC, NEPS, and the MC suggest higher probabilities to participate in AET for people in West Germany, the AME based on the AES indicates a higher probability for people in the east. This also applies after excluding AET activities that are not vocational. Interestingly, while the probabilities for east and west in the other surveys converge, in the AES the gap between east and west is widening.

With respect to gender, the results for overall AET based on AES and PIAAC do not show gender specific differences. In contrast, in the NEPS and the MC the probabilities for women are higher than for men. With focus on participation in vocational AET the proportions in the AES and PIAAC shift toward a disadvantage for women. The AMEs based on NEPS and the MC still indicate slightly higher probabilities for women.

Based on estimates in AES, PIAAC and the MC the effects point to a significant under-representation of older people (ages 55–64) in AET compared to younger people. This result is also stable if only vocational AET activities are taken into account.

Results regarding the relationship between education and AET participation reflect previous findings according to which the probability of participation increases with higher educational attainment. Within all surveys the results show a substantial gap between the groups of low-qualified (levels 0–2) and highly-qualified (levels 5A and 6). With focus on vocational AET this gap slightly reduces within the four surveys.

Likewise, there are no substantial differences between the surveys in comparison of the impact of employment on participation in AET. Based on all of the four surveys the AMEs indicates much lower probabilities for people who are not employed. With vocational AET this gap becomes even wider.

In summary, we determine different participation structures for (vocational AET) on the basis of harmonized representative samples and identical models. While education and employment show the same relationship over the surveys, we cannot make a clear statement for region, gender, and age about participation opportunities related to these characteristics.

## Discussion: TOWARD a True Value of Participation in Adult Education and Training?

Lifelong learning has become ever more prominent, especially in light of digitalization, rapid changes in professional activities and work contexts, as well as the active adult life spans continuously being longer. Accordingly, there should be a high level of interest in the ability to reliably monitor and analyze AET backed by a solid database and valid indicators. However, there is large variation in AET participation rates across surveys. Our research was designed to identify reasons for the present shortcomings and to contribute to improving the use of AET statistics so that they might better serve as secondary data in educational research and monitoring. We drew on the TSE as an overall framework and applied it to four surveys.

### Key Findings and Consequences for Educational Research

A first analysis showed that the four surveys (AES, PIAAC, NEPS, MC) were comparable in key points in regards to representation and measurement of AET. More detailed analyses on their implementation, however, revealed several differences. As far as representation is concerned, we find the most noticeable differences in voluntary (PIAAC, AES, NEPS) or mandatory (MC) survey participation. They also vary depending on whether the units are determined by the use of population registers (PIAAC, AES, MC) or by random route procedures (AES). Our results on measurement show differences in how the questionnaires support the interpretation of AET and the recall of AET events with a relatively high support of recall in AES and PIAAC in comparison to MC and yet another approach in the NEPS with its episode-assisted measurement. Overall, our initial analysis shows that varying AET results cannot be traced back to just one decisive factor but a variety of details and potential interacting error sources.

Our empirical comparisons addressed sample characteristics, participations rates, and participation patterns. After harmonizing the samples, their characteristics show similar distributions of key socio-demographic variables and only a slight educational bias in the voluntary surveys in comparison to the MC. A minor shift in participation rates can be detected before and after applying survey weights. After weighing and harmonizing the samples, the participation rates show a substantial gap of around 30 (±3) percentage points for overall AET and vocational AET participation rates.

Beyond comparing participation rates, we studied the participation patterns for both types of AET by conducting a multivariate analysis. Both overall and vocational AET show similar patterns for employment status and educational qualification across the surveys. On the one hand, it is reassuring that our analysis of survey results confirms the core statements of empirical educational research, most prominently the Matthew effect that refers to higher participation chances for those who already attained higher initial education (Boeren, 2009). On the other hand, the findings do yield differences between the surveys that concern the quality of the association between AET and region, age, and gender. While according to the AES, persons in East Germany have an increased chance of participating in vocational AET compared to persons from West Germany, in NEPS and MC it is exactly the other way around; according to PIAAC there are no differences. We also identified a gender-specific difference according to which women have lower chances than men to participate in vocational AET in PIAAC, but higher chances in NEPS and MC; according to AES data, gender does not matter. Finally, we identified age related differences of a pattern in NEPS and PIAAC corresponds less and in AES and MC more to a negative linear relationship between vocational AET participation and increasing age.

What do these results indicate for the use of AET statistics as secondary data in educational research and monitoring? For example, disparities in living conditions between East and West Germany have been the focus of reporting since reunification. Depending on the data basis, however, in the case of AET participation we arrive at different results regarding regionally specific participation chances. To be on the safe side, we could only communicate findings that can be replicated when analyzing various data bases. This approach considerably limits the analysis' potential due to the low level of standardization and risks not meeting the requirements placed on empirical educational research. Creating transparency about uncertainties would be desirable. While this is constitutive in the context of research, it is not always feasible in the context of policy and monitoring. Meaningful indicators and evidence-oriented education policy can only absorb a certain level of uncertainty. We see the solution in the further development of the survey-based measurement of AET and in our study we find starting points, which we explain further after having explained the limitations of our approach.

### Limitations

The TSE approach was instrumental in conceptually decomposing the AET surveys as it allowed us to identify potential error sources, which can help explain variances in the surveys' AET statistics. The available data, however, neither allows us to quantify individual error components nor to consider their possible interactions. While our analysis contributes to assessing the quality of AET statistics, it does not allow us to assess the true value of AET participation. Even if this were possible, a comprehensive evaluation of AET statistics must go beyond the question of presence or absence of errors and account for different user perspectives and the purposes for which statistics were calculated (Groves and Lyberg, 2010, p. 861–867). This user- or purpose oriented perspective points to unavoidable trade-offs between over- or underestimating AET against the analytical potential, e.g., the longitudinal design of the NEPS, identifying differentiated subgroups within the large MC sample, the details on AET activities in the AES, or the information on skills and workplace requirements as an important context for AET in PIAAC. Economically, of course, an evaluation of the surveys should also include collecting information on cost-benefit ratios.

When it comes to the relationship between AET and key socio-demographic variables, depending on the data basis, we were able to show that participation in AET models partially reflect relationships that vary in strength or direction. However, this modeling does not allow us to assess whether deviations between the surveys are random or significant in the statistical sense. It furthermore needs to be added that these models were not primarily developed theoretically but that they are guided by the data's availability and comparability. Thus, our results can only cover a small part of the information provided by the surveys. The examination of other key variables, such as the participation volume or the relationship with other central AET participation predictors, e.g., characteristics of occupational activity, showed too little similarity in measurement to be considered in our comparative analysis.

A final limitation of our survey examination we would like to point out is that we were exclusively concerned with questions related to survey implementation and measurement and that the underlying theoretical AET concepts were of no concern to us. We derived our (vocational) AET definition from the operationalization of the surveys, which to a large extent are based on the CLA. Our study was not concerned with the extent to which this classification and its implementation align to established, related, or contested concepts in this field, such as education, training, learning, adulthood, or vocation (Tight, 2002, p. 12–36). That notwithstanding our results would certainly be essential for a comprehensive and multidimensional AET assessment.

### Further Research and Further Developing Adult Education and Training Statistics

The literature on AET statistics reveals that their comparability is limited, thus curtailing our efforts to gain a sound understanding of AET levels and developments. Our analysis illustrates differences in how AET is represented and measured and how this contributes to cross-survey inconsistencies. One main objective of monitoring in the field of AET statistics is to provide information on changes in participation rates. Given this, differences in absolute participation rates may be negligible. However, comparisons of developments in participation rates in AES and LFS in Europe show survey specific AET participation trends as well (Behringer and Schönfeld, 2014, p. 395; Dohmen et al., 2019). In order to examine this in more detail, further comparative research with longitudinal or repeated cross-sectional data sets on AET is necessary.

A desirable goal for the long-term development of AET statistics would be a theoretically sound and empirically tested item set for AET-related information accepted by researchers as well as practitioners that can be used as standard in all surveys (including international surveys). This would not only considerably improve our knowledge on current AET levels and developments but also complement efforts in survey research to standardize (national) and harmonize (cross-national) demographic and socio-economic variables (Hoffmeyer-Zlotnik, 2016). In addition to improved measurement, this would also provide improved connectivity between the surveys and their various focal points and strengths. Ultimately, significant developments in this field require the cooperation of multiple stakeholders in research and politics, in some cases on an international level. In this final section, we would like to point out some starting points for how research can contribute to this goal.

From a TSE perspective, AET representation and measurement could be further investigated and improved best within designs in which central survey components alternate in order to detect error properties (Groves and Lyberg, 2010, p. 874). These can be complex undertakings that require a high degree of cooperation between different actors (sponsors, survey statisticians, and survey institutes, educational researchers) and often contradict the objectives intended by repeated cross-sectional surveys. However, smaller projects could also contribute in a complementary manner.

With regard to errors of representation, for example, efforts in non-response analysis (Halbesleben and Whitman, 2013) could indicate whether interest in AET, participation in AET and participation in the survey are related and therefore one-topic surveys might overestimate participation rates due to a non-response bias.

Studies focusing on the measurement of AET require slightly less effort and costs and can be carried out in smaller research projects. Based on our findings we recommend a multi-method approach that combines different evaluation methods for survey questions (The Federal Committee on Statistical Methodology, 2016), e.g., cognitive interviews or focus groups (Bowden et al., 2002; Lenzner et al., 2016). The results can inform how respondents interpret the survey questions on AET and how they recall and report AET activities. These results in turn allow to derive hypotheses and test them in survey experiments (Fowler, 2004). For example, our findings indicate that examining gender-specific interpretations and response behavior in more detail could considerably contribute to better understanding how (vocational) AET activities are measured best.

Evaluating AET measurements should also include conceptual considerations. Do the surveys' concepts cover, today and in the future, adult learning in a digitalized world and an ever-changing work context? Although meeting those criteria holds many challenges, it is necessary to pay tribute to them at least to some extent otherwise AET statistics will lag behind future developments by default (Felstead et al., 2005). Of course we understand that further technical and conceptual developments of AET measurements will lead to breaks in previously (more or less) continuous time series of repeated surveys. The trade-off then lies between either to being able to generate uninterrupted AET development statistics or to being able to generate up-to-date AET activity data.

There is an added layer of complication when it comes to adjusting international comparative AET surveys to current developments, as they require cross-national coordination processes. The literature points out that international comparability—also within the framework of the same survey—is severely limited by different national implementations (Hefler et al., 2010, p. 10; Cedefop, 2015, p. 30–37; Kaminska and Lynn, 2017). Thus, country differences in AET participation not only reflect the impact of national frameworks but also differences in measurement, sample design, and data quality. Here, according to the TSE paradigm, the same principles apply when comparing different national surveys; intersocietal differences between the target population, however, result in another error component (Smith, 2011).

The integration of such projects in calls for tender for AET surveys can create incentives to conduct this type of research more systematically and resource-supported. In this way, policymakers could support a systematic research programme and, ultimately, the further development of continuing training statistics.

## Data Availability Statement

Publicly available datasets were analyzed in this study. This data can be found here: PIAAC http://dx.doi.org/10.4232/1.12660, AES doi: 10.4232/1.11822, NEPS doi: 10.5157/NEPS:SC6:9.0.1, Microcensus RDC of the Federal Statistical Office and Statistical Offices of the Länder in Germany. This paper uses data from the National Educational Panel Study (NEPS): Starting Cohort Adults, doi: 10.5157/NEPS:SC6:9.0.1. From 2008 to 2013, NEPS data was collected as part of the Framework Program for the Promotion of Empirical Educational Research funded by the German Federal Ministry of Education and Research (BMBF). As of 2014, NEPS is carried out by the Leibniz Institute for Educational Trajectories (LIfBi) at the University of Bamberg in cooperation with a nationwide network.

## Ethics Statement

Ethical review and approval was not required for the study on human participants in accordance with the local legislation and institutional requirements. Written informed consent for participation was not required for this study in accordance with the national legislation and the institutional requirements.

## Author Contributions

BG and SW contributed with the initial conceptualization of the study. Each author was responsible for information on and data preparation of a specific survey: SW for AES, BG and NM for PIAAC, MH for NEPS, and JC for MC. JC supervised the joint analysis of the data and contributed the data presentation. NM and BG contributed a draft to section Comparative analysis of adult education and training participation and participation patterns in four population-based surveys. JC contributed a draft to section Results. The remainder of the article was contributed by SW. All authors contributed to the revision of the final draft.

### Conflict of Interest

The authors declare that the research was conducted in the absence of any commercial or financial relationships that could be construed as a potential conflict of interest.

## Footnotes

^1^Changes in the questionnaire and other survey components are quite common in repeated surveys; all following specifications refer to the survey “as is” in the survey year closest to the reference period of this analysis, 2012.

^2^We estimate the models with Statas' logit command and svy-prefix. Therefore, we use the provided weights for each survey. As far as there are more complex weighting procedures suggested, we apply them in our analyses (also see Table S5).

^3^Data was restricted to cases without missing values. With exception of NEPS, the share of missing values per variable is <2% of the full sample of individuals aged 25 to 64 years across the surveys. In the NEPS, the share of missing values on vocational AET is 3.5% (also see Table S4).

^4^When comparing the participation rates in the NEPS, we have to account for a relatively high number of missings on vocational AET (3.5% of the full sample in ages 25–64; also see Table S4). An analysis of missings at the individual level showed no abnormalities with regard to the variables used in this study.

## Abbreviations

AES: adult education study
AET: adult education and training
AME: average marginal effect
CAPI: computer assisted personal interview
CATI: computer assisted telephone interview
CLA: classification of learning activities
ISCED: international standard classification of education
LFS: labour force survey
MC: microcensus
NEPS: national educational panel study
OECD: organization for economic co-operation and development
PIAAC: programme for the international assessment of adult competencies
SC: starting cohort
TSE: total survey error.
