# Supplementary material for: The Quality of Data on Participation in Adult Education and Training. An Analysis of Varying Participation Rates and Patterns Under Consideration of Survey Design and Measurement Effects
Source: Front Sociol. 2019 Nov 14;4:71. doi: 10.3389/fsoc.2019.00071 (PMC8022492; doi:10.3389/fsoc.2019.00071)
Supplement: Supplementary file 1 [file Table_1.DOCX]

**Table S1. Extended overview of survey design of AES, PIAAC, NEPS and MC.**

|  | **AES** | **PIAAC** | **NEPS (SC 6, wave 5)** | **MC** |
| --- | --- | --- | --- | --- |
| **Sponsorship** | Federal Ministry of Education and Research | Federal Ministry of Education and Research, Federal Ministry of Labour and Social Affairs | Federal Ministry of Education and Research | Federal Government and regional states |
| **Survey Institute** | Infratest (since 2016 known as Kantar Public) | Infratest (since 2016 known as Kantar Public) | infas | Statistical offices of the States (Länder) |
| **Study design** | Repeated cross-sectional study, every 2 – 3 years | Repeated cross-sectional study, every 10 years | Yearly panel-study | Repeated cross-sectional study, yearly, with a 4-year rotating panel |
| **Data collection method** | CAPI | CAPI | CAPI (87.5%), CATI (12.5%) | CAPI (76.8%)/self-administered paper questionnaire (20.8%)/CATI (2.4%) |
| **Fieldwork** | 03/2012 – 06/2012 | 08/2011 – 03/2012 | 10/2012 – 05/2013 | All through the year |
| **Target Population** | Residential population, living in private households in Germany, aged 18 to 64 | Residential population, living in private households in Germany, aged 16 to 65 | Residential population, living in private households in Germany, birth cohorts 1944 – 1986 | Residential population, living in private households and shared accommodations in Germany aged 0 and over (questions on AET: aged 15 and over) |
| **Language of questionnaire** | German | German | German, Russian, Turkish | German |
| **Sampling frame and design** | stratified multilevel random sample, 1,300 sample points, random route procedure, selection key for respondent in household | population registry-based, two-stage stratified and clustered random sample, 320 sample points | population registry-based, stratified multilevel random sample, approx. 260 sample points | population registry-based single-stage stratified cluster sample with a sampling fraction of 1% of persons and households, 49 200 sample units; every year one quarter of the sampling units is replaced, practically every respondent participates for four years in a row if they do not move residence |
| **Sample size** | 7,099 respondents, 16,322 target persons (gross sample size) | 5,465 respondents, 10,240 target persons (gross sample size) | 11,696 respondents, 15,249 target population | 688,900 respondents  de facto anonymized data for scientific use covers 70% of the sample (511,946 including 476,342 principally residents) |
| **Obligation** | voluntary | voluntary | voluntary | mandatory, penalty payment |
| **Response rate** | 49.7% | 55% (incl. design weights) | 76.7% | 97.9% of households |
| **Recruitment of/contact to respondents** | Doorstep | Advanced letter, information material and monetary incentive | Advanced letter and monetary incentive | Official notice in advance |
| **Interest/Topic**  **Survey is introduced as** | …a scientific study; topic “adult learning” is introduced upon respondents’ request according to interviewers instructions | …a study on adults skills | …a scientific study on adults education and lifelong learning | …survey on the population and the labor market |
| **Interviewer** | No information | 5-day-training, analysis of interviewer effects | 1-day (CATI) to 5-day training (CAPI) | No information |
| **Weighting procedure**  **includes …** | …design adjustment and redressment to parameters: region, age, gender, school qualification, employment, German/not German, according to the update of latest population census and MC 2010 | …design, nonresponse adjustment and redressment to parameters: region, age, gender, school qualification, employment according to the population count by the Federal Statistical Office | …design adjustment and redressment to parameters: region, age, gender, school qualification, country of birth, according to MC 2012 | …nonresponse adjustment and redressment to parameters: region, age, gender, citizenship, according to the update of latest population census and central register of foreign nationals |
| **Sources; Quality reports** | Bilger et al., 2013; Infratest, 2013 | Rammstedt, 2013; Zabal et al. 2014 | Blossfeld et al. 2011; Blossfeld et al. 2016; Hammon et al., 2016; FDZ-LIfBi 2019; Bech et al. 2013 | Destatis, 2013 |

**Table S2. Extended overview of measurement of AET in the AES, PIAAC, NEPS and MC.**

|  | **AES** | **PIAAC** | **NEPS (SC 6, wave 5)** | **MC** |
| --- | --- | --- | --- | --- |
| **Introduction to AET questions** | There is a wide range of educational and further training opportunities for adults. This list contains various examples, divided into four types of event.  We are interested to know whether you have participated in such [show card] educational or further training events in the last 12 months for professional or personal reasons. | Now it’s about other courses and training you may have attended in the last 12 months either for professional or non-professional reasons. These are courses like on the show card.  A course conducted through open or distance education  An organized session for on-the-job training or training by supervisors or co-workers  A seminar or workshop  Other kind of course or private lesson | Minor variations over episodes:  Now I have a few questions about the courses and seminars you have attended during <for example civilian service, this activity, parental leave, ...> last year.  Out of episodes:  Let's get back to the subject of training. So far we have not noted any courses or training courses that you have attended since the last interview  We are also interested in courses and seminars that you have made for yourself, e.g. by attending a cooking course, a language course or a trainer course. | Have you participated in general or vocational training in the last 12 months? |
| **Reference period for AET participation** | 12 months previous to interview | 12 months previous to interview | Time span since the last interview | 12 months previous to interview |
| **Support of recall** | Show card with a list of four kind of activities [and specific vocational or non-vocational examples]  A Courses and training courses in work or leisure [Business English/Political Education Course]  B Short-term educational events: Lectures, training courses, seminars, workshops [management training/ natural history guided tour]  C Training / on-the-job training by supervisors, colleagues, trainers, teletutors [induction/trainee programs]  D Private lessons in leisure time [driving school/music lessons] | Show card with a list of four kind of activities  Have you participated in  distance learning in the last 12 months - in traditional or e-learning form?  training courses in the last 12 months within the scope of your professional activity or have you been trained by superiors or colleagues?  workshops or seminars during the last 12 months?  In the last 12 months, have you taken OTHER courses or private lessons that you have NOT mentioned? | Recall within specific contexts (see above) | Further explanation:  Forms of further education are for example courses, seminars, training courses, conferences, private lessons, study circles.  Vocational further education is retraining, training courses or courses for professional advancement, for new professional tasks, further education (computer, management, rhetoric, etc.).  General continuing education courses are usually for private purposes and serve to acquire or expand one's own skills and knowledge (music, sport, education, health, art, politics, technology, cooking, etc.). |
| **Number of courses and selection process** | 12 AET activities, further loops for up to 4 and up to 2 (randomly selected) activities | 1 AET activity, most recent | 2 (randomly selected) AET activities | Global questions for all resp. last activity reported |
| **Measurement of vocational AET** | Did you participate mainly for professional reasons or more for private reasons?  Professional / private | Have you attended this course or training mainly for professional reasons?  yes / no | In the following, we want to learn a bit more about the courses you attended in the last year. Let's begin with the course <h_kursbez>. Did you attend this course primarily for professional reasons or rather out of personal interest? for professional reasons / for private interest / (both)^a^ | What was the purpose of your training?  Professional / private / both professional and private |
| **Panel conditioning** | First time respondents | First time respondents | Panel respondents familiar with questionnaire | First time respondents (25%) and panel respondents familiar with questionnaire (75%) |
| **Placement in Questionnaire (roughly)** | in the middle of the interview | at the beginning of the interview | mainly within reported episodes, varies over respondents | towards the end of the interview; |
| **Proxy interviews** | No | No | No | 26% |
| **Sources** | Infratest, 2013 | Gesis, ohne Jahr | LIfBi, 2016 | Statistische Ämter, 2012 |
| *^a^According to the manual, "both" are not read out as an answer category and are rather an option for the interviewer if the respondent answers correspondingly.*  *The questions are translated using the online machine learning translation service DeepL to ensure a certain degree of standardization and objectivity; essential excerpts are reproduced here.* | | | | |

**Table S3. Extended Operationalization of vocational AET in the AES, PIAAC, NEPS and MC.**

|  | **AES** | **PIAAC** | **NEPS (SC 6, wave 5)** | **MC** |
| --- | --- | --- | --- | --- |
| **Information to identify vocational AET from up to** | 12 AET activities | 1 AET activity, most recent | 2 (randomly selected) AET activities | Global questions for all activities reported |
| **Measurement of vocational AET** | Did you participate mainly for professional reasons or more for private reasons? | Have you attended this course or training mainly for professional reasons? | Have you attended this course for professional or private reasons? | What was the purpose of your training? |
| **Values** | **1 = professional**  2 = private | 0 = no  **1= yes** | **1 =for professional reasons**  2= for private interest  **3 = both^a^** | **1 = professional**  2 = private  **3 = both professional and private** |
| **activities automatically assigned to vocational AET** | Training/on-the-job training by supervisors, colleagues, trainers, teletutors | Training courses within the scope of your professional activity or have you been trained by superiors or colleagues |  |  |
| *^a^According to the questionnaire instructions, the category "both" does not seem to be actively used in the interview.*  *The questions are translated using the online machine learning translation service DeepL to ensure a certain degree of standardization and objectivity.* | | | | |

**Table S4. Missing values on analysed variables.**

|  | **AES** | **PIAAC** | **NEPS** | **MC** |
| --- | --- | --- | --- | --- |
| **Full sample:  individuals aged 25 to 64 (N)** | **6,213** | **4,350** | **10,428** | **258,444** |
| overall AET | - | 78 (1.79%) | - | 288 (0.11%) |
| vocational AET | 2 (0.03%) | 79 (1.82%) | 366 (3.51%) | 288 (0.11%) |
| gender | - | - | - | - |
| age | - | - | - | - |
| region | - | - | 13 (0.12%) | - |
| ISCED-97 | 5 (0.08%) | 82 (1.89%) | 11 (0.11%) | 497 (0.19%) |
| employment status | 1 (0.02%) | 78 (1.79%) | - | - |
| *Sources: AES 2012; PIAAC 2012; NEPS SC 6, wave 5; Microcensus 2012 (own calculations).* | | | | |

**Table S5. Applied weighting procedures (Stata).**

| **AES** | svyset [pw=pgew] |
| --- | --- |
| **PIAAC** | svyset [pw=SPFWT0], jkrw(SPFWT1-SPFWT80, mult(`=79/80') reset) vce(jackknife) |
| **NEPS** | svyset psu [pweight=w_t5_cal], strata(stratum) singleunit(certainty) |
| **MC** | svyset [pw=EF952] |

**Table S6. Determinants of participation in overall AET (weighted logistic regressions): average marginal effects with 95% confidence intervals.**

|  | **AES** | **PIAAC** | **NEPS** | **MC** |
| --- | --- | --- | --- | --- |
| **gender** |  |  |  |  |
| male | ref | ref | ref | ref |
| female | 0.007 [-0.021; 0.034] | -0.005 [-0.033; 0.023] | 0.065 [0.036; 0.094] | 0.019 [0.016; 0.021] |
| **age** |  |  |  |  |
| 25-34 | ref | ref | ref | ref |
| 35-44 | -0.040 [-0.081; 0.001] | 0.017 [-0.038; 0.072] | 0.016 [-0.034; 0.066] | -0.015 [-0.019; -0.010] |
| 45-54 | -0.054 [-0.093; -0.014] | -0.025 [-0.071; 0.021] | -0.013 [-0.059; 0.033] | -0.029 [-0.033; -0.025] |
| 55-64 | -0.121 [-0.164; -0.079] | -0.144 [-0.188; -0.099] | -0.040 [-0.089; 0.010] | -0.076 [-0.080; -0.072] |
| **region** |  |  |  |  |
| west | ref | ref | ref | ref |
| east | 0.035 [0.001; 0.069] | -0.037 [-0.080; 0.006] | -0.060 [-0.095; -0.025] | -0.022 [-0.025; -0.019] |
| **ISCED-97** |  |  |  |  |
| 0-2 | -0.389 [-0.442; -0.336] | -0.465 [-0.534; -0.395] | -0.302 [-0.359; -0.246] | -0.244 [-0.249; -0.239] |
| 3+4 | -0.229 [-0.265; -0.193] | -0.243 [-0.281; -0.205] | -0.198 [-0.232; -0.164] | -0.167 [-0.171; -0.162] |
| 5B | -0.040 [-0.087; 0.006] | -0.085 [-0.139; -0.032] | -0.092 [-0.139; -0.044] | -0.026 [-0.033; -0.020] |
| 5A+6 | ref | ref | ref | ref |
| **employment status** |  |  |  |  |
| employed | ref | ref | ref | ref |
| unemployed and others | -0.247 [-0.278; -0.215] | -0.258 [-0.293; -0.223] | -0.141 [-0.179; -0.103] | -0.122 [-0.125; -0.119] |
| **N** | **6,207** | **4,268** | **10,404** | **257,783** |
| *Average marginal effects with 95% confidence intervals; ref=reference category.*  *Sources: AES 2012; PIAAC 2012; NEPS SC 6, wave 5; Microcensus 2012 (own calculations).* | | | | |

**Table S7. Determinants of participation in overall AET (weighted logistic regressions): predictive margins with 95% confidence intervals.**

|  | **AES** | **PIAAC** | **NEPS** | **MC** |
| --- | --- | --- | --- | --- |
| **gender** |  |  |  |  |
| male | 0.482 [0.462; 0.501] | 0.505 [0.479; 0.530] | 0.323 [0.302; 0.344] | 0.159 [0.157; 0.161] |
| female | 0.489 [0.470; 0.508] | 0.500 [0.475; 0.524] | 0.388 [0.365; 0.411] | 0.177 [0.175; 0.179] |
| **age** |  |  |  |  |
| 25-34 | 0.538 [0.507; 0.569] | 0.536 [0.497; 0.575] | 0.366 [0.325; 0.407] | 0.196 [0.193; 0.199] |
| 35-44 | 0.498 [0.471; 0.525] | 0.553 [0.516; 0.590] | 0.382 [0.349; 0.415] | 0.181 [0.179; 0.184] |
| 45-54 | 0.485 [0.461; 0.509] | 0.511 [0.477; 0.545] | 0.353 [0.328; 0.377] | 0.167 [0.164; 0.169] |
| 55-64 | 0.417 [0.388; 0.446] | 0.392 [0.362; 0.422] | 0.326 [0.297; 0.355] | 0.120 [0.117; 0.122] |
| **region** |  |  |  |  |
| west | 0.478 [0.463; 0.493] | 0.510 [0.488; 0.532] | 0.367 [0.348; 0.387] | 0.172 [0.170; 0.174] |
| east | 0.513 [0.482; 0.543] | 0.473 [0.432; 0.514] | 0.307 [0.278; 0.337] | 0.150 [0.147; 0.153] |
| **ISCED-97** |  |  |  |  |
| 0-2 | 0.277 [0.234; 0.321] | 0.227 [0.169; 0.286] | 0.201 [0.151; 0.251] | 0.052 [0.049; 0.055] |
| 3+4 | 0.437 [0.419; 0.455] | 0.450 [0.422; 0.477] | 0.305 [0.284; 0.327] | 0.129 [0.128; 0.131] |
| 5B | 0.626 [0.590; 0.661] | 0.607 [0.566; 0.648] | 0.411 [0.376; 0.447] | 0.270 [0.265; 0.275] |
| 5A+6 | 0.666 [0.636; 0.696] | 0.692 [0.657; 0.728] | 0.503 [0.472; 0.534] | 0.296 [0.292; 0.300] |
| **employment status** |  |  |  |  |
| employed | 0.545 [0.529; 0.562] | 0.564 [0.543; 0.586] | 0.378 [0.359; 0.397] | 0.193 [0.191; 0.194] |
| unemployed and others | 0.299 [0.273; 0.325] | 0.306 [0.270; 0.342] | 0.237 [0.204; 0.270] | 0.071 [0.069; 0.073] |
| **N** | **6,207** | **4,268** | **10,404** | **257,783** |
| *Predictive margins with 95% confidence intervals.*  *Sources: AES 2012; PIAAC 2012; NEPS SC 6, wave 5; Microcensus 2012 (own calculations).* | | | | |

**Table S8. Determinants of participation in vocational AET (weighted logistic regressions): average marginal effects with 95% confidence intervals.**

|  | **AES** | **PIAAC** | **NEPS** | **MC** |
| --- | --- | --- | --- | --- |
| **gender** |  |  |  |  |
| male | ref | ref | ref | ref |
| female | -0.022 [-0.048; 0.005] | -0.039 [-0.068; -0.010] | 0.027 [0.002; 0.052] | 0.014 [0.011; 0.017] |
| **age** |  |  |  |  |
| 25-34 | ref | ref | ref | ref |
| 35-44 | -0.050 [-0.090; -0.010] | 0.009 [-0.043; 0.062] | 0.034 [-0.009; 0.078] | -0.012 [-0.016; -0.008] |
| 45-54 | -0.050 [-0.089; -0.012] | -0.037 [-0.084; 0.010] | 0.011 [-0.030; 0.051] | -0.027 [-0.031; -0.023] |
| 55-64 | -0.125 [-0.167; -0.084] | -0.147 [-0.192; -0.103] | -0.036 [-0.078; 0.006] | -0.074 [-0.079; -0.070] |
| **region** |  |  |  |  |
| west | ref | ref | ref | ref |
| east | 0.051 [0.019; 0.083] | -0.011 [-0.048; 0.026] | -0.034 [-0.064; -0.005] | -0.018 [-0.021; -0.014] |
| **ISCED-97** |  |  |  |  |
| 0-2 | -0.340 [-0.392; -0.288] | -0.428 [-0.491; -0.366] | -0.226 [-0.278; -0.175] | -0.234 [-0.239; -0.229] |
| 3+4 | -0.214 [-0.249; -0.179] | -0.219 [-0.257; -0.181] | -0.169 [-0.201; -0.137] | -0.160 [-0.164; -0.155] |
| 5B | -0.033 [-0.079; 0.012] | -0.066 [-0.122; -0.010] | -0.095 [-0.139; -0.050] | -0.023 [-0.030; -0.017] |
| 5A+6 | ref | ref | ref | ref |
| **employment status** |  |  |  |  |
| employed | ref | ref | ref | ref |
| unemployed and others | -0.327 [-0.355; -0.299] | -0.317 [-0.351; -0.284] | -0.220 [-0.246; -0.193] | -0.130 [-0.133; -0.127] |
| **N** | **6,205** | **4,267** | **10,038** | **257,783** |
| *Average marginal effects with 95% confidence intervals; ref=reference category.*  *Sources: AES 2012; PIAAC 2012; NEPS SC 6, wave 5; Microcensus 2012 (own calculations).* | | | | |

**Table S9. Determinants of participation in vocational AET (weighted logistic regressions): predictive margins with 95% confidence intervals.**

|  | **AES** | **PIAAC** | **NEPS** | **MC** |
| --- | --- | --- | --- | --- |
| **gender** |  |  |  |  |
| male | 0.432 [0.413; 0.451] | 0.454 [0.431; 0.478] | 0.255 [0.236; 0.274] | 0.153 [0.151; 0.154] |
| female | 0.410 [0.392; 0.429] | 0.415 [0.391; 0.439] | 0.282 [0.262; 0.301] | 0.167 [0.164; 0.169] |
| **age** |  |  |  |  |
| 25-34 | 0.476 [0.446; 0.507] | 0.475 [0.435; 0.514] | 0.265 [0.230; 0.299] | 0.186 [0.182; 0.189] |
| 35-44 | 0.427 [0.401; 0.452] | 0.484 [0.451; 0.517] | 0.299 [0.270; 0.328] | 0.174 [0.171; 0.176] |
| 45-54 | 0.426 [0.403; 0.449] | 0.438 [0.406; 0.469] | 0.275 [0.252; 0.299] | 0.159 [0.156; 0.161] |
| 55-64 | 0.351 [0.323; 0.380] | 0.327 [0.298; 0.357] | 0.229 [0.203; 0.255] | 0.111 [0.109; 0.114] |
| **region** |  |  |  |  |
| west | 0.411 [0.397; 0.426] | 0.438 [0.418; 0.458] | 0.275 [0.258; 0.292] | 0.163 [0.161; 0.164] |
| east | 0.462 [0.433; 0.491] | 0.427 [0.392; 0.462] | 0.241 [0.216; 0.266] | 0.145 [0.142; 0.148] |
| **ISCED-97** |  |  |  |  |
| 0-2 | 0.246 [0.203; 0.288] | 0.176 [0.122; 0.230] | 0.167 [0.120; 0.213] | 0.048 [0.045; 0.050] |
| 3+4 | 0.371 [0.354; 0.389] | 0.385 [0.360; 0.409] | 0.224 [0.205; 0.242] | 0.122 [0.120; 0.123] |
| 5B | 0.552 [0.517; 0.587] | 0.538 [0.494; 0.582] | 0.298 [0.266; 0.330] | 0.258 [0.253; 0.263] |
| 5A+6 | 0.586 [0.556; 0.615] | 0.604 [0.569; 0.639] | 0.393 [0.364; 0.422] | 0.281 [0.277; 0.285] |
| **employment status** |  |  |  |  |
| employed | 0.499 [0.483; 0.516] | 0.509 [0.486; 0.531] | 0.302 [0.285; 0.318] | 0.186 [0.184; 0.187] |
| unemployed and others | 0.172 [0.150; 0.194] | 0.191 [0.163; 0.220] | 0.082 [0.059; 0.104] | 0.056 [0.054; 0.058] |
| **N** | **6,205** | **4,267** | **10,038** | **257,783** |
| *Predictive margins with 95% confidence intervals.*  *Sources: AES 2012; PIAAC 2012; NEPS SC 6, wave 5; Microcensus 2012 (own calculations).* | | | | |
